# Stroke patients’ knowledge about cardiovascular family history - the Norwegian Stroke in the Young Study (NOR-SYS)

**DOI:** 10.1186/s12883-015-0276-6

**Published:** 2015-03-12

**Authors:** Halvor Øygarden, Annette Fromm, Kristin Modalsli Sand, Geir Egil Eide, Lars Thomassen, Halvor Naess, Ulrike Waje-Andreassen

**Affiliations:** Department of Clinical Medicine, University of Bergen, Bergen, Norway; Department of Neurology, Haukeland University Hospital, N-5021 Bergen, Norway; Centre for Clinical Research, Haukeland University Hospital, Bergen, Norway; Lifestyle Epidemiology Research Group, Department of Global Public Health and Primary Care, University of Bergen, Bergen, Norway

**Keywords:** Young stroke, Family history, Ischemic stroke, Cardiovascular disease

## Abstract

**Background:**

Family history (FH) is a risk factor for cardiovascular disease, especially coronary artery disease (CAD). The impact on risk of stroke is less clear. This study investigated young and middle-aged ischemic stroke patients’ knowledge on FH of stroke, CAD, and peripheral artery disease (PAD) with a special regard to sex differences.

**Methods:**

From September 2010 to February 2014, all ischemic stroke patients aged 15–60 years were prospectively included in the Norwegian Stroke in the Young Study (NOR-SYS). FH of stroke, CAD and PAD in offspring, siblings, parents, and grandparents was assessed using a standardized face-to-face interview. In addition to ‘yes’ and ‘no’, the optional reply ‘don’t know’ was included to improve accuracy. McNemar’s test was used to compare paired proportions, i.e. FH in male vs. female relatives. Multiple logistic regression analyses were used to test the influence of patient sex on FH reporting and to adjust for possible confounding factors.

**Results:**

Altogether 257 patients were included. Mean age was 49.5 years and 68.1% were males. FH of cardiovascular disease was reported by 59% of patients. When asked about FH of stroke, 48 (18.7%) and 46 (17.9%) patients reported yes, whereas 17 (6.6%) and 9 (3.5%) reported ‘don’t know’ regarding father and mother respectively, similarly patients reported ‘don’t know’ regarding 117 (45.5%) paternal vs. 83 (32.4%) maternal grandmothers (p < 0.001). Female patients reported less ‘don’t know’ and were more likely to report a positive cardiovascular FH than males (OR: 3.4; 95% CI: 1.5 to 7.7; p = 0.004). Patients had more detailed knowledge about CAD than stroke in fathers (p < 0.001), mothers (p < 0.001) and siblings (p = 0.01).

**Conclusions:**

Young and middle-aged stroke patients reported a high FH burden of cardiovascular disease. Females are more likely to report a positive FH than males. Detailed knowledge on FH was best for CAD. Our results suggest sex has a big impact on FH knowledge. Females have more knowledge of FH than males and knowledge is better for relatives with a female than male linkage.

**Clinical trial registration:**

http://www.clinicaltrials.gov, unique identifier: NCT01597453.

## Background

Family history (FH) of cardiovascular disease (CVD) in first-degree relatives (FDRs), including stroke, coronary artery disease (CAD) and peripheral artery disease (PAD), is a risk factor for vascular disease [1,2]. The association between CAD and FH of CVD is well documented [3-6]. However, the impact on risk of ischemic stroke is less clear, although FH of CVD is a positive predictor of stroke risk [7-10]. Sibling and genetic studies support FH of CVD as a risk factor and suggest a genetic influence on ischemic stroke risk [11-13]. Females with stroke are more likely to have a positive parental history than are males, and females are also more likely to have a positive maternal than paternal history [14,15]. Why females are more likely to report a positive FH is unknown [16]. Earlier studies of FH in stroke patients seldom separate between FH of intracranial hemorrhage (ICH) and ischemic stroke, assuming that it would be difficult for patients to differentiate between these [7-9,17-19]. Few studies included a reply of ‘don’t know’ regarding FH, and when included it was usually interpreted as negative, to avoid over-estimating the FH burden [7,9]. However, one study found that 11% answered ‘don’t know’ regarding FH in FDRs [20]. According to Flossman et al., publications on genetic epidemiology of stroke are heterogeneous, insufficiently detailed and possibly biased [8]. Today, more detailed information about CVD and risk factors is available for patients and their family members. Diagnostic stroke imaging has improved and increased detection of stroke [21]. Repeated efforts to increase awareness of acute stroke symptoms have been implemented after the introduction of thrombolytic therapy [22]. Therefore the public should be better qualified than ever to give a precise account of their FH. As we enter the genomic era of medicine, FH still is the most accessible, inexpensive and well proven tool assessing inherited risk for disease [23].

This population-based study, performed in a well-defined region of western Norway, aims to explore what young and middle-aged ischemic stroke patients know about stroke, CAD and PAD in their families. We aimed to assess and quantify a detailed FH of CVD with a special regard to sex differences.

## Methods

Ischemic stroke patients aged 15–60 years who were prospectively included in the population-based Norwegian Stroke in the Young Study (NOR-SYS) were assessed. The methods and rationale of NOR-SYS have been described in detail previously [24]. Acute cerebral infarction was documented by magnetic resonance imaging. Patients unable to provide an adequate FH due to severe stroke, aphasia or severe psychiatric illness, and patients who were adopted or had no contact with their biological family were excluded. This single-center study has Hordaland County as catchment area, from which all patients aged up to 60 years with suspected stroke are admitted to the stroke unit at Haukeland university hospital.

Patients were interviewed using a standardized questionnaire within day two or three after the diagnosis of acute ischemic stroke. The interview was done face to face to ensure the patient did not contact family members by mobile phone or in any other way during the interview; and to ensure only events recalled by the patient were registered. All registration of events was done by the interviewing doctor. To increase reproducibility of answers between study doctors, new interviewers participated as a bystander in at least 5 interviews, thereby increasing the interview similarity and minimizing differences in answer interpretation. Data regarding patient sex, age, education, number of siblings and offspring were registered in addition to a detailed disease history and family history. Patients were assigned to the educational categories, basic school, high school or college/university education. The three optional replies for the FH disease entities of stroke, heart disease and PAD/claudication were: ‘yes’, ‘no’ and ‘don’t know’. ‘Don’t know’ was included to improve accuracy of reporting. The frequency of ‘don’t know’ in FH was also analyzed to assess the effect of patient sex on reporting of FH. In addition, the frequency of ‘don’t know’ was analyzed to assess differences in reporting of maternal vs. paternal FH. The frequency of the answer ‘don’t know’ regarding type of heart and cerebrovascular disease in FDRs was analyzed to compare patients’ knowledge on FH of heart disease with their knowledge on cerebrovascular disease. All reported non-CAD, if present without any CAD, was regarded as no CAD. FH of stroke, heart disease and PAD in FDRs; parents, siblings and biological offspring was registered. In addition, FH of all four grandparents was registered.

To explore in depth knowledge and avoid misinterpretation of disease, further questions were asked. If the patient replied ‘yes’ regarding FH of any of the disease entities stroke, heart disease and PAD, he was asked to specify the type of stroke or heart disease and in case of PAD he was asked to specify the prescribed treatment. When stroke was reported, the patient was asked to specify the type of stroke as a Transient Ischemic Attack (TIA)/minor stroke with quick and complete restitution, cerebral infarction, ICH or ‘don’t know’. When heart disease was reported, this was specified as ischemic, such as angina pectoris and myocardial infarction, as non-CAD, such as arrhythmia, valve problem and heart insufficiency or ‘don’t know’. If PAD/claudication was reported, the patient was asked about the applied treatment, such as training, surgical treatment other than amputation, amputation or ‘don’t know’. If one answer for disease subtype was missing, the data was imputed as ‘don’t know’.

### Statistics

Descriptive statistics are given using the mean, standard deviation (SD) and proportion with 95% confidence interval (CI). The chi square test was used for categorical data. McNemar’s test was used to compare paired proportions. Continuous and normally distributed variables were analyzed by Student’s *t*-test. Wilcoxon’s Rank-Sum Test was used to analyze continuous variables that were not normally distributed.

We stratified the patients by sex to compare FH between males and females. FH of CVD was considered present if at least one parent, sibling or grandparent had CVD. Multiple logistic regression analyses with FH of CVD as dependent variable and age, sex, educational category and, to adjust for family size, number of siblings as independent variables were performed. The same analyses were performed using FDRs only, to ensure that the high rates of ‘don’t know’ regarding grandparental FH did not affect the main results. The level of significance was set at 0.05. Stata 13.1 (StataCorp, College Station, TX) was used for all analyses.

### Ethics

All patients or legal guardians signed a written informed consent. NOR-SYS is approved by the Regional Ethics Committee of western Norway, and the study was conducted in accordance with the Declaration of Helsinki.

## Results

### Demographics

Between September 2010 and February 2014, 292 stroke patients were included in NOR-SYS. Two patients did not consent, and two others were not included because of serious psychiatric illness and mental retardation. Thirty-five patients were excluded after inclusion. Three (1%) were adopted and had no contact with their biological families and 32 (10.1%) were unable to answer for themselves due to severe aphasia or coma. Participants had a mean age of 49.5 (SD = 9.3) years, 68% were male and the majority had at least a high school education (76%, Table 1). The mean number of siblings was 2.5 and 237 (92%) patients had at least one sibling. No offspring stroke, CAD or PAD were reported. There were no significant differences in demographic data by sex, however there was a trend for age (p = 0.057), females were slightly younger than males (47.6 years vs. 50.4 years, respectively).

**Table 1:** **Demographic data of the 257 patients included in the Stroke in the Young Study (NOR-SYS) in Bergen, Norway 2010-2014**

**Variables**	**Total N = 257**	**Males N = 175 (68.1%)**	**Females N = 82 (31.9%)**	**P**
Age in years, *mean* (*SD*)	49.5 (9.3)	50.4 (8.5)	47.6 (10.6)	0.057
Education				0.547
Basic school, n (%)	60 (23.5)	43 (24.7)	17 (21.0)	
High school, n (%)	91 (35.7)	64 (36.8)	27 (33.3)	
College/University, n (%)	104 (40.8)	67 (38.5)	37 (45.7)	
N of siblings, *mean* (*SD*)	2.5 (1.7)	2.6 (1.9)	2.2 (1.3)	0.116
N of children, *mean* (*SD*)	2.0 (1.3)	2.0 (1.4)	1.8 (1.2)	0.311
Deceased fathers, *n* (%)	149^*^ (58.7)	103 (59.9)	46 (56.1)	0.756
Deceased mothers, *n* (%)	92^†^ (36.4)	60 (35.1)	32 (39.0)	0.542
First-degree FH of stroke, n (%)	87 (33.9)	55 (31.4)	32 (39.0)	0.230
First-degree FH of CAD, n (%)	105 (41.0)	67 (38.3)	38 (46.9)	0.192
First-degree FH of PAD, n (%)	16 (6.2)	11 (6.3)	5 (6.1)	0.954

*Abbreviations*: *SD* standard deviation, *FH* family history, *First-degree FH* family history in parents, siblings or offspring, *CAD* coronary artery disease, *PAD* peripheral artery disease, *P* p-value of comparison between males and females.

^*^N = 254 due to missing data in 5 of patients’ fathers. ^†^N = 252 due to missing data in 4 of patients’ mothers.

### Family history

About 59% of participants reported their father was deceased, while 36% reported a deceased mother (p < 0.001, Table 1). Two patients did not know if their fathers were alive, and data regarding deceased parents was missing in four patients. Any first-degree FH of CVD was reported by 153 (59.5%) patients. Most participants reported a first-degree FH of CAD (41%), followed by stroke (34%) and PAD (6%). Patients reported relatively low numbers of disease and high proportions of ‘don’t know’ in grandparents for all types of CVD (Table 2).

**Table 2:** **Reported family history of cardiovascular disease in first-degree relatives** ^*^ **and grandparents of the 257 patients included in the Stroke in Young Study (NOR-SYS) in Bergen, Norway 2010–2014**

**Relatives**	**Cardiovascular disease**	**Yes N (%)**	**No N (%)**	**Don’t know N (%)**
Siblings^†^	Stroke	8 (3.3)	232 (95.9)	2 (0.8)
	Heart disease	36 (14.9)	202 (83.5)	4 (1.6)
	PAD	5 (2.1)	232 (95.9)	5 (2.1)
Mothers	Stroke	46 (17.1)	202 (78.6)	9 (3.5)
	Heart disease	55 (21.6)	187 (73.3)	13 (5.1)
	PAD	9 (3.5)	233 (90.7)	15 (5.8)
Fathers	Stroke	48 (18.7)	192 (74.7)	17 (6.6)
	Heart disease	101 (39.3)	141 (54.9)	15 (5.8)
	PAD	15 (5.8)	221 (86.0)	21 (8.2)
Mothers’ mothers	Stroke	30 (11.7)	143 (55.9)	83 (32.4)
	Heart disease	35 (13.7)	126 (49.2)	95 (37.1)
	PAD	1 (0.4)	180 (70)	76 (29.6)
Mothers’ fathers	Stroke	16 (6.2)	142 (55.5)	98 (38.3)
	Heart disease	47 (18.3)	113 (44.0)	97 (37.7)
	PAD	3 (1.2)	173 (67.3)	81 (31.5)
Fathers’ mothers	Stroke	20 (7.8)	120 (46.7)	117 (45.5)
	Heart disease	21 (8.2)	121 (47.1)	115 (44.7)
	PAD	5 (1.9)	167 (65.0)	85 (33.1)
Fathers’ fathers	Stroke	18 (7.0)	114 (44.4)	125 (48.6)
	Heart disease	34 (13.2)	108 (42.0)	115 (44.8)
	PAD	4 (1.6)	156 (60.7)	97 (37.7)

*Abbreviations*: *Stroke* both ischemic events and intracranial hemorrhage, *Heart disease* including coronary artery disease and reported non-CAD such as heart failure, rhythm and/or valve problems, *PAD* peripheral artery disease.

^*^No cardiovascular events were reported among offspring.

^†^N = 242, 242 patients had one or more siblings.

### FH knowledge regarding type of CVD

Patient reports on type of CVD in FDRs are summarized in Table 3. Patients reported more CAD among fathers than among mothers (p < 0.001). Detailed knowledge regarding type of heart disease was high, whereas knowledge on type of parental stroke was lower. Comparing answers regarding disease type, ‘don’t know’ type of stroke was significantly higher than ‘don’t know’ type of heart disease for fathers (p < 0.001), mothers (p < 0.01), siblings (p = 0.01), and all grandparents (p < 0.001) except mothers’ fathers (p = 0.5). Few patients reported a FH of PAD and the knowledge of ordinated treatment for PAD was not analyzed further.

**Table 3:** **Comparing knowledge on type of cardiovascular disease in patients replying ‘Yes’ a family member suffered from stroke, heart disease and/or peripheral artery disease**

	**Siblings** ^*^	**Mothers**	**Fathers**	**Mothers’ mothers**	**Mothers’ fathers**	**Fathers’ mothers**	**Fathers’ fathers**
**Stroke**	**N = 8**	**N = 46**	**N = 48**	**N = 30** ^**†**^	**N = 16**	**N = 20**	**N = 18** ^**†**^
TIA (%)	0 (0.0)	9 (19.6)	8 (16.7)	4 (13.3)	3 (18.8)	2 (10.0)	0 (0.0)
Cerebral infarction (%)	3 (37.5)	12 (26.1)	11 (22.9)	2 (6.7)	4 (25)	2 (10.0)	2 (11.1)
Cerebral bleeding (%)	1 (12.5)	7 (15.2)	5 (10.4)	1 (3.3)	2 (12.5)	1 (5.0)	0 (0.0)
Don’t know (%)	4 (50)	18 (39.1)	24 (50)	23 (76.7)	7 (43.8)	15 (75.0)	16 (88.9)
**Heart disease**	**N = 36**	**N = 55** ^**†**^	**N = 101** ^**†**^	**N = 35** ^**†**^	**N = 47** ^**†**^	**N = 21**	**N = 34** ^**†**^
Angina pectoris (%)	5 (13.9)	12 (21.8)	9 (8.9)	8 (22.9)	4 (8.5)	4 (19.1)	3 (8.8)
Myocardial infarction (%)	15 (41.7)	16 (29.1)	48 (47.5)	8 (22.9)	24 (51.1)	8 (38.1)	16 (47.1)
Non-CAD (%)	12 (33.3)	21 (39.6)	25 (24.8)	7 (20)	3 (6.4)	2 (9.5)	2 (5.9)
Don’t know (%)	4 (11.1)	6 (10.9)	19 (18.8)	12 (34.3)	16 (34.0)	7 (33.3)	13 (38.2)
**PAD**	**N = 5** ^**†**^	**N = 9** ^**†**^	**N = 15**	**N = 1**	**N = 3**	**N = 5**	**N = 4**
Conservative (%)	2 (40.0)	3 (33.3)	2 (13.3)	0 (0.0)	0 (0.0)	1 (20.0)	0 (0.0)
Revascularization surgery (%)	2 (40.0)	1 (11.1)	5 (33.3)	1 (100)	0 (0.0)	0 (0.0)	0 (0.0)
Amputation (%)	0 (0.0)	0 (0.0)	2 (13.3)	0 (0.0)	3 (100)	2 (40.0)	1 (25.0)
Don’t know (%)	1 (20.0)	5 (55.6)	6 (40)	0 (0.0)	0 (0.0)	2 (40.0)	3 (75.0)

From the 257 patients included in the Stroke in Young Study (NOR-SYS) in Bergen, Norway 2010-2014.

*Abbreviations*: *TIA* transient ischemic attack, *Non-CAD* non-coronary heart disease, included heart failure, rhythm and/or valve problems.

^*^N = 242, 242 patients had one or more siblings.

^†^One answer regarding type of disease was missing and was imputed as don’t know.

### Sex differences in FH

**Figure 1:**
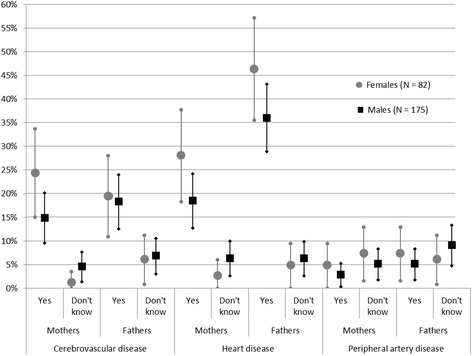
**Reported parental history of cerebrovascular disease, coronary artery disease and peripheral artery disease from the 257 young and middle-aged ischemic stroke patients included in the Stroke in the Young Study (NOR-SYS) in Bergen, Norway 2010–2014, stratified by sex.** Answers ‘Yes’ and ‘Don’t know’ are displayed in percentage proportions with 95% confidence intervals of the total N, the remaining answering ‘No’.

There was a trend of more reported FH of CVD events and less frequent reporting of ‘don’t know’ among females compared to males (Figure 1). When analyzing FH of CVD in grandparents, females reported significantly less ‘don’t know’ regarding heart disease in mothers‘ mothers (p = 0.02) and of stroke in fathers’ mothers (p = 0.02). Patients reported ‘Yes’ or ‘No’ on FH of stroke in both parents in 235 (91%) cases and having knowledge of both parents’ FH was most common. Patients consistently reported less ‘don’t know’ regarding maternal FH than paternal FH (Table 4). Males reported a mean number of 1.5 (SD: 1.28) family members with CVD, whereas females reported 1.9 (SD: 1.25; p = 0.01). Females reported a positive FH more often than males with an OR of 3.4 (95% CI: 1.5 to 7.7; p < 0.01; Table 5). When analyzing a positive FH in FDRs only, females are more likely to report a positive FH with an OR of 2.5 (95% CI: 1.3 to 4.8; p < 0.01). In addition, increasing age was associated with a positive FH and higher educational category was associated with a negative FH.

**Table 4:** **Comparing patients answering ‘don’t know’ regarding family history of cardiovascular disease in maternal vs. paternal family members**

**Relatives**	**Maternal**	**Paternal**	
**CVD**	**N (%)**	**N (%)**	**P** ^*^
Parent			
Stroke	9 (3.50)	17 (6.61)	0.059
CAD	13 (5.06)	15 (5.84)	0.512
PAD	15 (5.84)	21 (8.17)	0.157
Grandfathers			
Stroke	98 (38.13)	125 (48.64)	0.000
CAD	81 (31.52)	97 (37.74)	0.006
PAD	97 (37.74)	115 (44.75)	0.024
Grandmothers			
Stroke	83 (32.30)	117 (45.53)	0.000
CAD	95 (36.96)	115 (44.75)	0.000
PAD	76 (29.57)	85 (33.07)	0.117

From the 257 patients included in the Stroke in the Young Study (NOR-SYS) in Bergen, Norway 2010-2014.

*Abbreviations*: *CVD* Cardiovascular disease, *Stroke* both ischemic events and intracranial hemorrhage, *CAD* coronary artery disease, *PAD* peripheral artery disease.

^*^P-value calculated with McNemar’s test.

**Table 5:** **Logistic regression displaying factors possibly associated with a positive family history of cardiovascular disease of the 257 patients included in the Stroke in the Young Study (NOR-SYS) in Bergen, Norway 2010-2014**

**Response variable:**	**FH of FDR**			**FH of FDR + grandparents**		
**Predictors**	**OR**	**95% CI**	**P**	**OR**	**95% CI**	**P**
Sex (female)	2.50	(1.31, 4.78)	0.005	3.37	(1.48, 7.70)	0.004
Education	0.67	(0.47, 0.98)	0.038	1.20	(0.79, 1.80)	0.377
Number of siblings	1.08	(0.9, 1.27)	0.329	0.99	(0.83, 1.18)	0.922
Age of patient (years)	1.09	(1.0, 1.13)	<0.001	1.03	(0.99, 1.06)	0.116

*Explanations and abbreviations*: *FH* family history, *FDR* first-degree relatives, i.e. parents, siblings and offspring (no cardiovascular events were reported for offspring in this study), *OR* Odds ratio, *CI* Confidence interval, *Education* basic school, high school and college/university: Number of siblings: 1 unit increase per sibling reported.

## Discussion

We observed a high rate of reported CVD in patients’ parents. The reported FH of parental stroke in the present study was 33%, slightly lower than 41% reported in a Swedish study [9]. The reported 37% FH of CAD in the present study is comparable with 38% in the Swedish study [9]. The slight disparity regarding parental stroke may be explained by the lower mean age of our patients and the methodological differences concerning the acquisition of FH, where we solely interviewed patients. The present study showed higher numbers of deceased fathers than mothers (149 vs. 92), probably caused by the higher life expectancy of females [25].

Earlier studies have observed a mother-daughter relationship in heredity of stroke [14]. Our results show that females are more likely to report a positive FH than males. Females reported a higher incidence of FH of CVD in total and had better knowledge on the type of CVD. In addition, both male and female patients know more about their maternal than paternal FH of CVD. Both sex of patient and maternal family linkage influence the response rate of ‘don’t know’; this suggests that knowledge of FH is strongly influenced by sex, possibly due to females being more involved in communication across generations in Norway. Additionally, the cultural designation of females as main family care-givers may enable them to obtain more information on FH of CVD [26].

Significant less reporting of ‘don’t know’ regarding disease type in maternal vs. paternal grandparents supports the hypothesis that female-female communication on disease across generations increases knowledge on FH. Another possible explanation for the higher maternal FH may be that males have a higher risk of violent death at young age, before CVD manifestations occur [27]. And the higher female reporting of FH may be explained by females with ischemic stroke simply having a higher FH of CVD burden than do males. However, these hypotheses do not explain the higher maternal than paternal FH also when comparing same-sex grandparents.

The patients reporting parental stroke had difficulties differentiating between the types of stroke, 40% and 50% of patients answered ‘don’t know’ regarding mothers’ and fathers’ type of stroke, respectively. The present study was conducted 20 years after the introduction of MRI and we assumed that the new diagnostic and treatment opportunities in addition to informational campaigns would have improved patients’ knowledge about stroke. Our reported numbers of parental ICH in relation to total stroke numbers were comparable to the relationship found in epidemiologic studies [28]. Patients seem to clearly recall a FH of ICH, but have more problems defining an ischemic stroke in their FH. This may be due to higher mortality and often more dramatic symptoms of ICH [29]. It is reported that general knowledge on stroke is lower than knowledge on CAD, although the knowledge about stroke symptoms was not lower in newer studies [30,31]. Less reporting of ‘don’t know’ on type of heart disease than on type of stroke regarding all FDRs in our study suggests less knowledge about stroke than CAD.

The reported FH of PAD was low in the present study; only 15 (5.8%) of patients’ fathers had PAD. In a recent Dutch study including 4700 patients with a history of cerebrovascular disease, CAD, PAD or aortic abdominal aneurysm, 16% of patients had a FH of PAD, and they found that paternal PAD was a risk factor for subsequent PAD in the offspring [32]. This difference may be caused by the lower mean age of our patients, and that only patients with ischemic stroke were included in our study. However, the difference in prevalence of FH shows the importance of addressing all manifestation sites of atherosclerosis and vascular disease when evaluating FH.

The reported FH of CVD among grandparents was low. The high reporting of ‘don’t know’ regarding grandparents’ FH of CVD and the almost absent knowledge of grandparents’ particular CVD type suggests this may be due to lack of knowledge on grandparental disease history and not absence of disease among grandparents. Less available medical care and less precise diagnostics may explain the lack of knowledge. In addition, the generation gap reduces information of grandparental FH.

The present study is strengthened by the homogenous and well-defined study population, and also the detailed assessment of FH including CVD subtypes and the analysis of FH from several generations. The study also has some limitations. The well-defined study population of young ischemic stroke patients makes the results not directly generalizable to the general population. In addition, the self-reported FH may not be completely correct and is dependent on family relations, the patient cognitive status at time of the interview and several other factors. We excluded patients with aphasia and patients incapable of answering themselves, but we did not assess if severity of the acute disease could influence the answers regarding FH. However, the inclusion of ‘don’t know’ as a possible answer increases the accuracy in providing a potential answer for patients unsure of their FH.

## Conclusion

In conclusion, the FH of CVD burden among young ischemic stroke patients was high. Females seem to have more knowledge on FH of CVD than do males, and knowledge on maternal FH is higher than paternal FH. Knowledge on FH of heart disease type is significantly higher than type of stroke. We recommend obtaining the FH with the patient as the primary informant, however, the involvement of other family members may increase both the completeness and accuracy of the FH and should be encouraged. More public information on FH of CVD as a risk factor is warranted to improve the general knowledge of FH in the population. Information could be directed towards males in particular. As males have less knowledge regarding own FH than females; they have the most to gain by improving their FH knowledge. Increased knowledge of own FH provides an opportunity to take action to reduce risk and may encourage patients into smoking cessation, regular exercise and adopting a healthier diet. Increased attention on FH is important for the patient and also the public in general to improve this accessible, well-proven and inexpensive tool both for risk stratification and in aiding future genetic research.
